# Ameliorative effects of pine bark extract on cisplatin-induced acute kidney injury in rats

**DOI:** 10.1080/0886022X.2017.1282871

**Published:** 2017-02-08

**Authors:** In-Chul Lee, Je-Won Ko, Sung-Hyeuk Park, Na-Rae Shin, In-Sik Shin, Yun-Bae Kim, Jong-Choon Kim

**Affiliations:** a BK21 Plus Team, College of Veterinary Medicine, Chonnam National University, Gwangju, Republic of Korea;; b College of Veterinary Medicine, Chungbuk National University, Cheongju, Republic of Korea

**Keywords:** Cisplatin, acute kidney injury, apoptosis, oxidative stress, pine bark extract, protective effect

## Abstract

**Objective:** This study investigated the dose–response effects of pine bark extract (PBE, pycnogenol^®^) on oxidative stress-mediated apoptotic changes induced by cisplatin (Csp) in rats.

**Materials and methods:** The ameliorating potential of PBE was evaluated after orally administering PBE at doses of 10 or 20 mg/kg for 10 days. Acute kidney injury was induced by a single intraperitoneal injection of Csp at 7 mg/kg on test day 5.

**Results:** Csp treatment caused acute kidney injury manifested by elevated levels of serum blood urea nitrogen (BUN) and creatinine (CRE) with corresponding histopathological changes, including degeneration of tubular epithelial cells, hyaline casts in the tubular lumen, and inflammatory cell infiltration (interstitial nephritis). Csp also induced significant apoptotic changes in renal tubular cells. In addition, Csp treatment induced high levels of oxidative stress, as evidenced by an increased level of malondialdehyde, depletion of the reduced glutathione (GSH) content, and decreased activities of glutathione *S*-transferase, superoxide dismutase, and catalase in kidney tissues. On the contrary, PBE treatment lowered BUN and CRE levels and effectively attenuated histopathological alterations and apoptotic changes induced by Csp. Additionally, treatment with PBE suppressed lipid peroxidation, prevented depletion of GSH, and enhanced activities of the antioxidant enzymes in kidney tissue.

**Conclusions:** These results indicate that PBE has a cytoprotective effect against oxidative stress-mediated apoptotic changes caused by Csp in the rat kidney, which may be attributed to both increase of antioxidant enzyme activities and inhibition of lipid peroxidation.

## Introduction

Cisplatin [*cis*-diamminedichloroplatinum(II), Csp], one of platinum derivatives, is widely used as an anti-neoplastic agent for the treatment of various types of cancer, including carcinomas, sarcomas, lymphomas, and germ cell tumors.^1^^,^^2^ However, extensive application of Csp in clinical practice, particularly at high doses and as repeated cycles of a treatment regimen, is limited by its undesirable side effects, including nephrotoxicity.^3^^,^^4^ The pathogenesis of Csp-induced nephrotoxicity is characterized by necrosis and apoptosis of renal tubular cells, referred to as acute renal injury.^5–7^ Many studies have reported that Csp causes apoptotic cell death in renal tubular cell, but the underlying mechanism has remained unclear. It has been postulated that the toxicity of Csp occurs via various mechanisms, including inhibition of protein synthesis, DNA damage, and mitochondrial injury, ultimately leading to programed cell death.^8^ Although the exact mechanisms of Csp-induced nephrotoxicity are not yet fully elucidated, several evidences suggest that reactive oxygen species (ROS) are involved in the toxicity of Csp.^9–11^ This platinum derivative causes increased generation of ROS, such as superoxide anion and hydroxyl radicals, and inhibits antioxidant enzyme activities.^12–14^ Csp treatment decreases kidney glutathione (GSH) contents and increases lipid peroxidation, which is closely associated with the acute kidney injury caused by Csp.^15^^,^^16^ Recently, it has been reported that antioxidants and free radical scavengers reduce Csp-induced oxidative apoptosis via the inhibition of ROS generation in rats.^2^^,^^17–20^ Thus, a combination of effective antioxidant agents may be an appropriate approach to minimizing the toxic side effects of Csp with preserving its chemotherapeutic efficacy.

Pine bark extract (PBE, pycnogenol^®^) is a standardized proprietary mixture of bioflavonoid extracts from the bark of the French marine pine (*Pinus maritima*). Currently, PBE is widely used in dietary supplements, multivitamins, and health products because of its potent antioxidant activity.^21^ The major constituents of PBE are condensed flavonoids and polyphenols, particularly monomeric and oligomeric units of catechin, epicatechin, and taxifolin. PBE is a potent antioxidant and acts as a free radical scavenger by reacting with both hydroxyl radical and singlet oxygen.^21^^,^^22^ It enhances the synthesis of antioxidant enzymes and regenerates vitamins C and E.^23^^,^^24^ These pharmacological activities of PBE can confer protection against various degenerative conditions, including diabetes, asthma, hypertension, and chemical-induced toxicity caused by oxidative stress.^21^^,^^23^^,^^25–28^ Recently, it has also been reported that a 5-day repeated oral dose of pycnogenol^®^ at 200 mg/kg/day prior to the administration of Csp has an antioxidant effect on Csp-induced kidney injury in rats.^29^ However, the potential effects of PBE on apoptotic changes in the kidneys and the dose–response effects of PBE during Csp intoxication have not been elucidated until now. The aim of this study was to evaluate the protective effect of PBE on oxidative stress-mediated apoptotic changes induced by Csp in rats, in view of the fact that PBE is known to possess antioxidant properties.

## Materials and methods

### Animals and environmental conditions

Thirty male Sprague–Dawley rats (aged 9 weeks) were obtained from a specific pathogen-free colony at Samtako Co. (Osan, Korea) and used after 1 week of quarantine and acclimation. Two rats per cage were housed in a room maintained at a temperature of 23 ± 3 °C and a relative humidity of 50 ± 10%, with artificial lighting from 08:00 to 20:00 and 13–18 air changes per hour. The rats were provided with tap water sterilized by ultraviolet irradiation and a commercial rodent chow (Samyang Feed, Wonju, Korea) *ad libitum*. The Institutional Animal Care and Use Committee of Chonnam National University approved the protocols for the animal study (CNU IAUCU-YB-2013-13), and the animals were cared for in accordance with the Guidelines for Animal Experiments of Chonnam National University.

### Test chemicals and treatments

Csp (CAS No. 15663–27-1) was purchased from Sigma-Aldrich Co. (St. Louis, MO), and PBE was purchased from Horphag Research Ltd. (Le Sen, France). All other chemicals were of the highest grade commercially available. The test chemicals were dissolved in sterilized normal saline and prepared immediately before the treatment. The application volumes of Csp (2 mL/kg body weight) and PBE (10 mL/kg body weight) were calculated based on the most recently recorded body weight of an individual animal. PBE was administered to rats by gavage once daily for 10 days at dose levels of 10 and 20 mg/kg/day. Csp (7 mg/kg) was injected to rats intraperitoneally on test day 5, 1 h after the PBE treatment, to induce kidney injury based on previous studies.^2^^,^^10^ All animals were sacrificed 24 h after last PBE treatment on test day 10.

### Experimental groups and dose selection

Twenty-four healthy male rats were randomly assigned to four experimental groups (*n* = 6 per group): (1) vehicle control, (2) Csp, (3) Csp + PBE 10, and (4) Csp + PBE 20. The dose of Csp was selected based on previous studies.^27^^,^^29^ The effective doses of PBE were based on our previous study and earlier reports.^23^^,^^25^^,^^28^ All animals were observed daily for mortality throughout the test period. The body weight of each rat was measured on test days 0, 2, 4, 6, 8, and 10.

### Necropsy and serum biochemical analysis

At the end of the experimental period, all treated animals were euthanized by carbon dioxide inhalation for blood sample collection 5 days after Csp treatment (test day 10). Blood samples were drawn from the posterior vena cava. Serum samples were collected within 1 h after the blood collection by centrifugation at 800 *g* for 10 min and stored at −80 °C until analysis. The kidney injury caused by Csp and the effect of PBE were evaluated by assessing the serum levels of blood urea nitrogen (BUN) and creatinine (CRE) using an autoanalyzer (Dri-chem 4000i, Fujifilm Co., Tokyo, Japan). The absolute and relative (organ-to-body weight ratio) weights of the kidney were measured for all rats.

### Histopathological examination

The removed right kidney was fixed in a 10% neutral buffered formalin solution for 2 weeks. The left kidney was quickly frozen in dry ice and stored at −80 °C for biochemical analysis. The fixed kidney tissues were embedded in paraffin, sectioned to 4 μm thickness, deparaffinized, and rehydrated using standard techniques. The extent of Csp-induced kidney injury and the effects of PBE were evaluated by assessing morphological changes in kidney sections stained with hematoxylin and eosin. All observations were made manually using a light microscope (Leica DM LB2; Leica, Wetzlar, Germany) with ×5, ×10, ×20, and ×40 objective lenses and a ×100 oil immersion lens, in a totally blinded manner.

### Immunohistochemical analysis for caspase-3

To evaluate the effects of PBE on Csp-induced apoptotic changes in the kidneys, we conducted immunohistochemistry for caspase-3, which plays a critical role as an executioner of apoptosis, using the avidin–biotin complex (ABC) method (VECTASTAIN Elite Rabbit IgG ABC Kit; Vector Laboratories, Peterborough, UK). The paraffin sections were deparaffinized, dehydrated, washed in phosphate-buffered saline (PBS, pH 7.5), and incubated for 10 min at room temperature with 10% goat serum to block nonspecific staining. Subsequently, the slides were incubated with a primary rabbit anti-rat caspase-3 antibody (1:200 dilution; Cell Signaling Technology, Danvers, MA). The sections were washed and then incubated with biotinylated secondary antibody at 37 °C for 1 h, followed by incubation with a streptavidin-peroxidase at room temperature for 1 h. After the incubation, the sections were washed with PBS and incubated with 0.05% diaminobenzidine chromogen for 10 min. The sections were counterstained with hematoxylin, rinsed in PBS to terminate the reaction, and then mounted with cover slips for microscopic examination (Leica). Images were captured, and the intensity of caspase-3 immunopositivity was measured using an image analysis program (i-Solution system, IMT, Daejeon, Korea).

### Determination of lipid peroxidation, reduced glutathione, and antioxidant enzymes

The frozen left kidney tissues were homogenized in a glass-Teflon homogenizer with 50 mM phosphate buffer (pH 7.4) to obtain 1:9 (w/v) whole homogenate. The homogenate was then centrifuged at 11,000 × *g* for 15 min at 4 °C to remove any cell debris, and the supernatant was used to measure malondialdehyde (MDA), a marker of lipid peroxidation, and GSH levels. The concentration of MDA was assayed by monitoring thiobarbituric acid reactive substance formation using the method of Berton et al.^30^ The GSH content was measured by the method of Moron et al.^31^ Antioxidant enzyme activities, including catalase (CAT), superoxide dismutase (SOD), glutathione reductase (GR), and glutathione *S*-transferase (GST), were determined using commercial assay kits (Cayman Chemical, Ann Arbor, MI). The total protein content was determined using bovine serum albumin as a standard.^32^

### Statistical analyses

Data are expressed as the mean ± standard deviation (SD), and all statistical comparisons were made using one-way analysis of variance, followed by Tukey–Kramer multiple comparison tests. The data were analyzed with GraphPad InStat ver. 3.0 (GraphPad Software, Inc., La Jolla, CA). Differences with *p* values of .05 or lower were considered statistically significant.

## Results

### Effects of PBE on clinical signs, body weight, and kidney weight

There was no treatment-related mortality in the animals treated with PBE and/or Csp during the study period. However, the rats treated with Csp showed treatment-related clinical signs including depression (*n* = 1), loss of facial fur (*n* = 2), diarrhea (*n* = 4), and piloerection (*n* = 4) after Csp treatment (data not shown). Csp treatment also caused a significant decrease in the body weight on test days 8 and 10 compared with that in the control group (Table 1). The absolute and relative kidney weights in the Csp-treated rats significantly increased compared with those in the control group. Although the body weights in the Csp + PBE groups were decreased, there were no statistical significances compared with that in the control group. Additionally, the kidney weights in the Csp + PBE groups decreased significantly in a dose-dependent manner compared with that in the Csp group.

**Table 1.** Body weight of male rats treated with Csp and/or PBE.

Items	Csp/PBE (mg/kg)			
	0/0	7/0	7/10	7/20
No. of rats	6	6	6	6
Day 0	314.1 ± 12.6^a^	318.9 ± 12.4	319.0 ± 10.4	316.7 ± 14.4
Day 2	326.0 ± 13.8	324.0 ± 11.9	321.1 ± 11.5	324.9 ± 17.1
Day 4	334.3 ± 12.2	332.4 ± 22.0	331.8 ± 6.9	334.2 ± 17.7
Day 6	343.8 ± 11.2	331.0 ± 22.2	328.2 ± 12.3	331.0 ± 18.7
Day 8	340.5 ± 17.4	307.1 ± 20.7^b^	313.4 ± 18.8	318.6 ± 20.3
Day 10	346.7 ± 19.5	282.8 ± 21.1^b^	304.7 ± 20.4	306.2 ± 19.2
Kidneys (g)	2.40 ± 0.116	2.73 ± 0.221^b^	2.62 ± 0.190	2.55 ± 0.184
Per body weight (%)	0.69 ± 0.040	0.96 ± 0.079^b^	0.85 ± 0.068^c^	0.83 ± 0.061^c^

a Values are presented as means ± SD.

^b^*p* < .05, *p* < .01 as compared with the control group.

^c^*p* < .05, *p* < .01 as compared with the Csp group.

### Effects of PBE on Csp-induced renal dysfunction

As shown in Table 2, the serum BUN (77.2 ± 24.20 *vs.* 19.3 ± 5.21, *p <* .01) and CRE (6.93 ± 1.81 *vs.* 0.32 ± 0.08, *p* < .01) levels in the Csp group markedly increased compared with those in the control group. The serum BUN (52.3 ± 18.53) and CRE (5.45 ± 1.47) levels in the Csp + PBE 10 group slightly decreased, but were not significantly different from the Csp group. However, the levels of BUN (36.1 ± 12.90, *p <* .01) and CRE (3.63 ± 1.67, *p* < .01) in the Csp + PBE 20 group significantly decreased compared with those in the Csp group.

**Table 2.** Serum biochemical values of male rats treated with Csp and/or PBE.

Items	Csp/PBE (mg/kg)			
	0/0	7/0	7/10	7/20
No. of rats	6	6	6	6
BUN (mg/dL)	19.3 ± 5.21	77.2 ± 24.20^b^	52.3 ± 18.53	36.1 ± 12.90^c^
CRE (mg/dL)	0.32 ± 0.08	6.93 ± 1.81^b^	5.45 ± 1.47	3.63 ± 1.67^c^

BUN: blood urea nitrogen; CRE: creatinine.

a Values are presented as means ± SD.

b *p* < .01 as compared with the control group.

c *p* < .01 as compared with the Csp group.

### Effects of PBE on Csp-induced histopathological changes in kidney tissues

**Figure 1.**
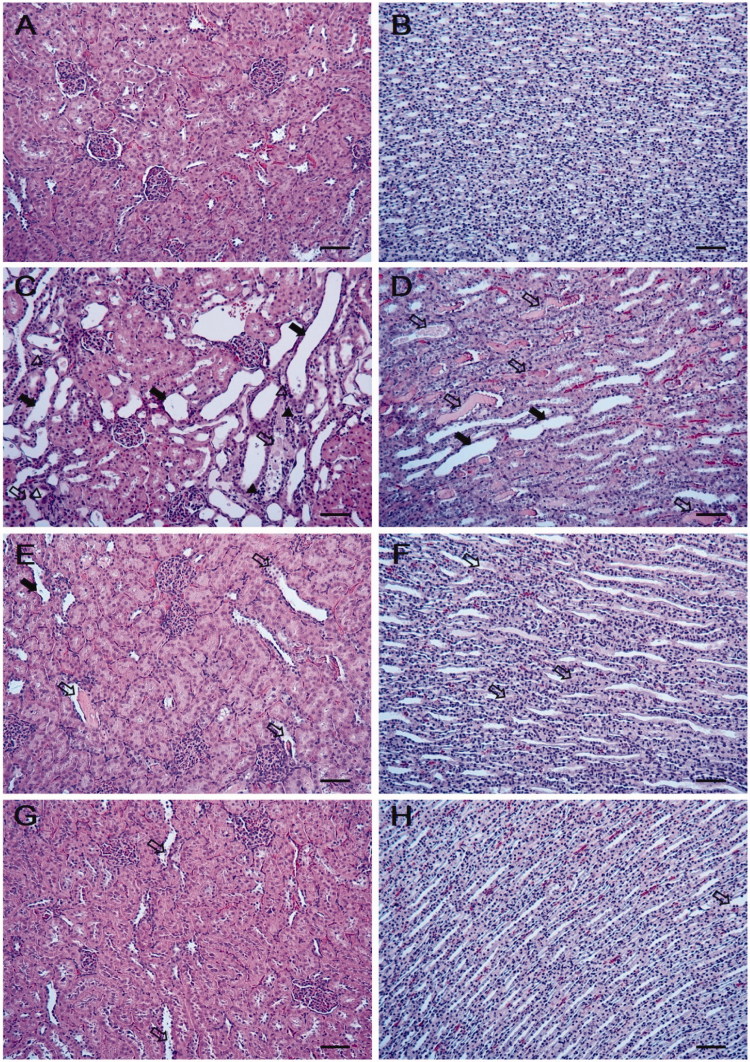
Effects of PBE on Csp-induced histological changes in kidney tissues. Representative images of kidneys from (A,B) vehicle control group, (C,D) Csp group, and (E–H) Csp + PBE groups. The kidneys from Csp-treated rats show moderate to severe desquamation of tubular epithelial cells (*closed arrows*), cell debris/hyaline cast in dilated tubular lumen (*open arrows*), inflammatory cell infiltration (*closed arrowheads*), and degeneration of tubular epithelial cells (*open arrowheads*). The kidneys from Csp + PBE groups show mild desquamation of tubular epithelial cells and cell debris/hyaline cast in dilated tubular lumen. Bar = 50 μm. Hematoxylin and eosin stain.

The control group presented kidneys with normal architecture (Figure 1(A,B)). However, the kidney tissue in the Csp group showed various histopathological alterations, including degeneration and desquamation of tubular epithelial cells, cell debris/hyaline cast in the dilated tubular lumen, and inflammatory cell infiltration (interstitial nephritis) (Figure 1(C,D)). Although these findings were also observed in the Csp + PBE groups, the treatment with PBE effectively decreased the incidence and severity of the Csp-induced histopathological lesions (Figure 1(E–H)).

### Effects of PBE on Csp-induced apoptotic changes in kidney tissues

**Figure 2.**
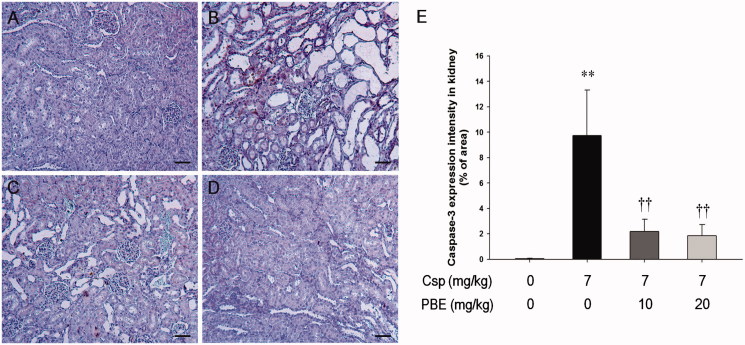
Effects of PBE on Csp-induced apoptotic changes in kidney tissues. Representative images of kidneys from (A) vehicle control group, (B) Csp group, and (C,D) Csp + PBE groups. Counterstaining with hematoxylin. Bar = 50 μm. (E) Immunopositivity for caspase-3 in kidneys from vehicle, Csp, and Csp + PBE groups. The values are presented as mean ± SD (*n* = 6). **p < .01 as compared with the control group. ††*p <* .01 as compared with the Csp group.

As presented in Figure 2, the control group had few caspase-3-positive cells in the kidney. The kidney tissue from the Csp-treated rats showed a marked increase in the number of caspase-3-positive cells or their intensity (Figure 2(B)). In contrast, the number of caspase-3-positive cells decreased in the kidneys from the Csp + PBE groups in a dose-dependent manner (Figure 2(C,D)). In addition, the intensity of caspase-3 expression also significantly decreased compared with that in the Csp group (Figure 2(E)).

### Effects of PBE on Csp-induced lipid peroxidation and depletion of GSH in kidney tissues

**Figure 3.**
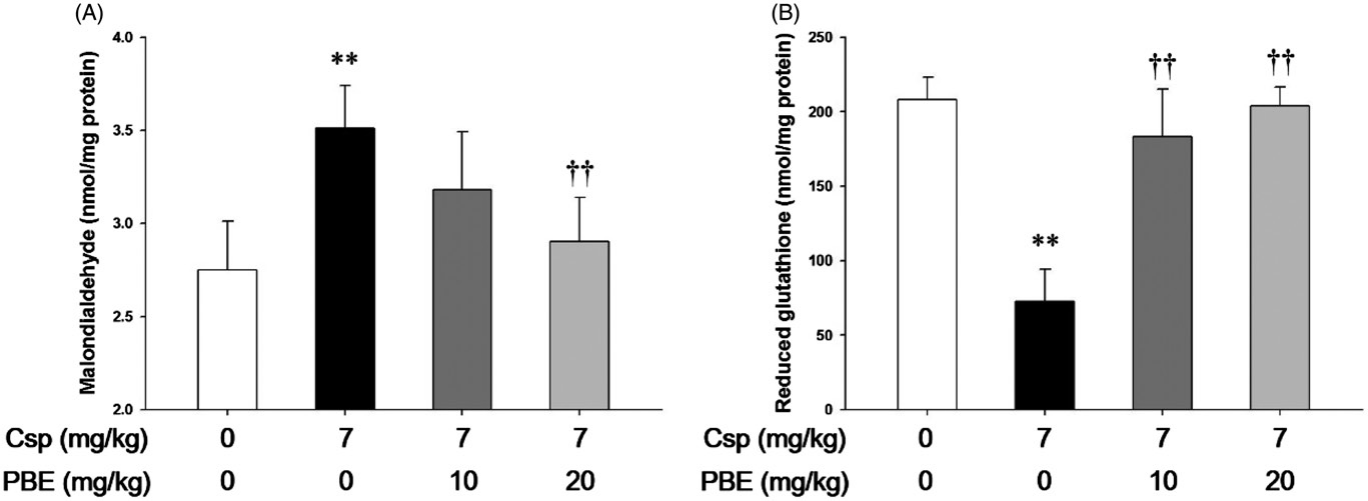
Effects of PBE on Csp-induced lipid peroxidation and depletion of reduced glutathione. The levels of (A) malondialdehyde, a marker of lipid peroxidation, and (B) reduced glutathione in kidney tissues treated with Csp or PBE. The values are presented as mean ± SD (*n* = 6). **p < .01 as compared with the control group. ††*p <* .01 as compared with the Csp group.

The concentration of MDA, an end product of lipid peroxidation, in the rats treated with Csp significantly increased (3.5 ± 0.23 *vs.* 2.8 ± 0.26, *p* < .01), but the GSH content significantly decreased (72.8 ± 21.29 *vs.* 208.3 ± 14.72, *p* < .01) compared with those in the control group, respectively (Figure 3). In the Csp + PBE 10 group, the kidney MDA content slightly decreased, but not statistically significant (3.1 ± 0.31 *vs.* 3.5 ± 0.23). The GSH content significantly increased compared with that in the Csp group (183.2 ± 32.20 *vs.* 72.8 ± 21.29, *p* < .01). In the Csp + PBE 20 group, the MDA level significantly decreased (2.9 ± 0.24 *vs.* 3.5 ± 0.23, *p* < .01), whereas the GSH content significantly increased (203.8 ± 13.01 *vs.* 72.8 ± 21.29, *p* < .01) compared with those in the Csp group.

### Effects of PBE on Csp-induced suppression of antioxidant enzyme activities in kidney tissues

**Figure 4.**
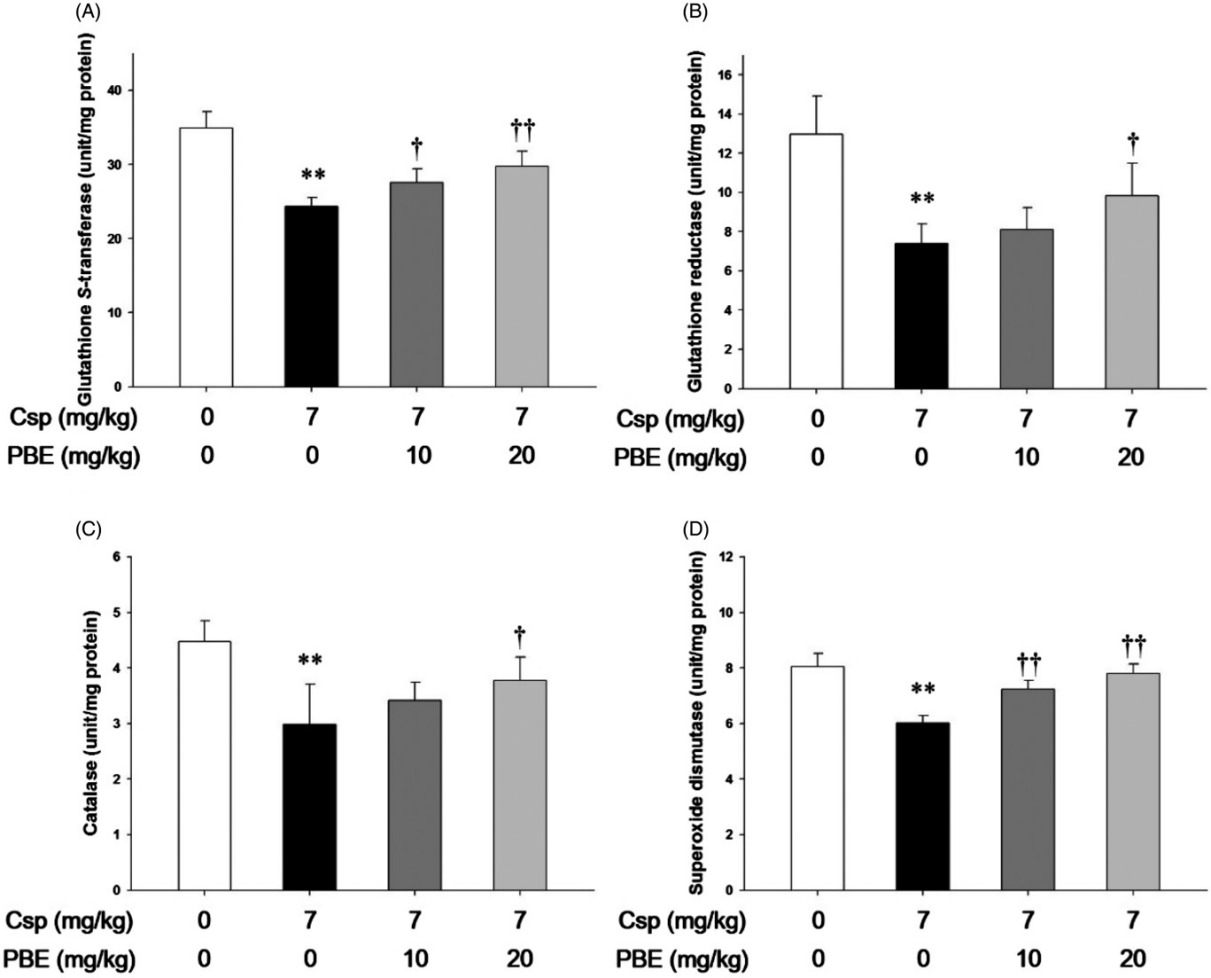
Effects of PBE on Csp-induced suppression of antioxidant enzyme activities. The levels of (A) glutathione *S*-transferase, (B) glutathione reductase, (C) catalase, and (D) superoxide dismutase in kidney tissues treated with Csp or PBE. The values are presented as mean ± SD (*n* = 6). **p < .01 as compared with the control group. †*p <* .05, ††*p <* .01 as compared with the Csp group.

As shown in Figure 4, the rats treated with Csp showed a significant decrease in the activity of GST (24.3 ± 1.19 *vs.* 34.9 ± 2.21, *p* < .01), GR (7.4 ± 0.98 *vs.* 12.9 ± 1.95, *p* < .01), CAT (2.9 ± 0.72 *vs.* 4.5 ± 0.38, *p* < .01), and SOD (6.0 ± 0.27 *vs.* 8.1 ± 0.48, *p* < .01) compared with those in the control group, respectively. The PBE treatment at 10 mg/kg/day resulted in a significant increase in the GST (27.6 ± 1.89 *vs.* 24.3 ± 1.19, *p* < .05) and SOD (7.2 ± 0.31 *vs.* 6.0 ± 0.27, *p* < .01) activities compared with those in the Csp group. The activities of CAT (3.4 ± 0.33) and GR (8.1 ± 1.12) slightly increased, but not significant different from the Csp group. In contrast, the treatment with PBE at 20 mg/kg/day significantly increased the activities of GST (29.8 ± 2.03 *vs.* 24.3 ± 1.19, *p* < .01), CAT (3.8 ± 0.41 *vs.* 2.9 ± 0.72, *p* < .05), GR (9.8 ± 1.65 *vs.* 7.4 ± 0.98, *p* < 0.05), and SOD (7.8 ± 0.33 *vs.* 6.0 ± 0.27, *p* < .01) compared with those in the Csp group.

## Discussion

The use of cancer chemotherapy is generally limited by undesirable side effects. Thus, strategies to minimize the side effects of chemotherapeutic agents, with preservation of their chemotherapeutic efficacy, are needed.^33^ One of the approaches to reduce the side effects of chemotherapy drugs is combined drug delivery together with potent antioxidant agents.^25^^,^^33^^,^^34^ PBE has been shown to confer ameliorative effects against various pathological conditions caused by oxidative stress.^25^^,^^26^^,^^28^ According to a previous study, Csp can generate highly reactive free radicals and interfere with the tissue antioxidant defense, which is considered to be a predominant cause of Csp nephrotoxicity.^11^ Even though PBE has been confirmed to have a protective effect against Csp-induced nephrotoxicity,^30^ the effects of PBE on Csp-induced oxidative kidney damage and apoptotic changes have not been fully elucidated. The results of this study indicate that PBE treatment provides a protective effect against Csp-induced oxidative stress and apoptotic changes by preserving the antioxidant enzyme activities and inhibiting lipid peroxidation in rats.

During the test period, the animals treated with Csp showed marked body weight loss. The weight loss observed in the Csp-treated rats may be attributed to gastrointestinal toxicity of Csp.^15^^,^^35^ This weight loss was not effectively prevented by the PBE treatment. Similar to other chemotherapy agents, chemotherapy with Csp causes many clinical adverse signs, particularly, hearing loss, hair loss, myelosuppression, emesis, and diarrhea.^36^^,^^37^ In the present study, clinical signs, including depression, diarrhea, and loss of facial fur, were observed during the study period in the Csp group, but these clinical signs were not recorded in the PBE-treated groups. This suggested that PBE effectively attenuated clinical symptoms caused by the Csp treatment.

Csp-induced kidney injury is recognized as the most common adverse effect and a dose-limiting factor associated with its clinical utility.^10^^,^^38^ Previous studies have demonstrated that Csp causes acute kidney injury, which is characterized by a change in the urine volume and an increase in the serum BUN and CRE levels.^5^^,^^15^ Our findings confirmed the results of previous studies showing that Csp treatment caused damage to the kidney with a marked elevation of serum BUN and CRE levels. The kidney damage caused by Csp was accompanied by corresponding histopathological changes, such as degeneration and desquamation of tubular epithelial cells, hyaline cast formation, inflammatory cell infiltration, and tubular dilation. These remarkable changes reflect that Csp treatment impairs kidney function and structure. In contrast, the PBE treatment effectively suppressed the serum BUN and CRE levels and attenuated the histopathological changes in kidney tissues, indicating that PBE confers protective effects against Csp-induced acute kidney injury. These findings are consistent with the results of a previous study showing that a 5-day repeated oral dose of pycnogenol^®^ (200 mg/kg/day) prior to the administration of Csp has an antioxidant effect on Csp-induced oxidative kidney injury in rats.^29^ In the present study, a 10-day repeated oral dose of PBE, before and after the administration of Csp, effectively prevented serum biochemical changes and histopathological alterations induced by Csp, even at much lower doses (10 and 20 mg/kg/day) of PBE. Thus, our findings indicate that treatment with PBE can confer a protective effect against Csp-induced acute kidney injury.

Despite the underlying mechanisms of Csp-induced nephrotoxicity are still unclear, ROS-mediated oxidative stress and apoptosis play important roles in the pathogenesis of acute kidney injury caused by Csp.^10^^,^^11^^,^^39^^,^^40^*In vivo* and *in vitro* studies using renal tubule epithelial cells provided evidence that Csp causes apoptotic changes via excessive generation of ROS.^19^^,^^41^ Csp treatment induces excessive production of free radicals, which cause lipid peroxidation and impairment of the redox status in renal tissues.^5^^,^^15^ In this study, a high degree of caspase-3 intensity was seen in kidney tubular epithelia of Csp-treated rats. In addition, Csp treatment caused high levels of oxidative stress in renal tissue, as evidenced by the increase of lipid peroxidation product MDA, depletion of the GSH content, and suppression of antioxidant enzyme activities in kidney tissues. Previous studies and our results clearly indicate that Csp-induced apoptotic changes in renal tubular cells are mediated by oxidative stress. However, treatment with PBE effectively attenuated apoptotic changes of renal tubular cells caused by Csp. Additionally, PBE treatment not only markedly suppressed the levels of MDA, but also increased the level of GSH and activities of GST, GR, SOD, and CAT in kidney tissues. In a number of previous studies, antioxidants or free radical scavengers have been shown to be effective in the protection from Csp-induced acute kidney injury.^12^^,^^15^^,^^16^^,^^42^ PBE acts as a free radical scavenger and confers protective effects by enhancing the endogenous levels of vitamins C and E, catalase, and GSH.^21–23^^,^^43^ The antioxidant activity of PBE attenuates various degenerative conditions caused by oxidative stress.^24^^,^^44^^,^^45^ Additionally, concomitant treatment with PBE attenuates the chemotherapy-induced adverse effects.^25^^,^^46^ The results of the previously mentioned studies and our data suggest that cytoprotective effects of PBE may be due to antioxidant activity, which is associated with the suppression of lipid peroxidation and restoration of antioxidant enzyme activities.

## Conclusions

Collectively, the results of this study indicate that PBE effectively attenuates Csp-induced acute kidney injury in rats. The ameliorating effects of PBE may be related to its ability to confer cytoprotection against oxidative stress-mediated apoptotic changes by inhibiting lipid peroxidation and by enhancing antioxidant enzyme activities. Since clinical exploitation with Csp carries a risk of acute kidney injury, these findings suggest that combination of PBE may be a potential therapeutic adjuvant against Csp-induced nephrotoxicity and various kidney injuries caused by oxidative stress.
